# Gene Expression Analysis of the Microdissected Trophoblast Layer of Human Placenta after the Spontaneous Onset of Labor

**DOI:** 10.1371/journal.pone.0077648

**Published:** 2013-10-17

**Authors:** Soo Hyun Kim, Sung Han Shim, Se Ra Sung, Kyung A. Lee, Jung Yun Shim, Dong Hyun Cha, Kyoung Jin Lee

**Affiliations:** 1 Department of Obstetrics and Gynecology, CHA Gangnam Medical Center, CHA University, Seoul, Republic of Korea; 2 Genetics Laboratory, Fertility Center of CHA Gangnam Medical Center, Seoul, Republic of Korea; 3 Department of Biomedical Science, College of Life Science, CHA University, Seoul, Republic of Korea; 4 Department of Pathology, CHA Gangnam Medical Center, CHA University, Seoul, Republic of Korea; Fudan University, China

## Abstract

**Background:**

Despite increasing evidence that human parturition is associated with alteration in gene expression in the uteroplacental unit, the precise mechanisms that elicit spontaneous term labor in humans remain unknown. Our goal in this study was to compare the mRNA expression pattern of the trophoblast layer of normal term placenta between women who had given natural birth (labor group) and those who had undergone an elective cesarean section without labor (non-labor group).

**Methods:**

We collected placental tissue samples from six pregnant women after term vaginal deliveries (labor group) and from six pregnant women after scheduled Cesarean sections (non-labor group). Frozen sections were made immediately after placental delivery. Because the placenta is a heterogeneous tissue composed of several cell types, we used laser capture microdissection to separate the trophoblast layer from the rest of the placental tissues.

**Results:**

A number of genes were differentially expressed in the trophoblast layer when the labor and non-labor groups were compared. The expression of *SIRT1*, *KAP1*, and *CRH* was significantly lower in the trophoblast layer of the labor group than of the non-labor group. The expression of *IL-1b*, *NF-kB1* and *TLR 8* in the labor group was significantly higher than that in the non-labor group.

**Conclusions:**

Human term labor may be closely associated with inflammatory response. We suggest that downregulation of *SIRT1*, *KAP1*, and *CRH* gene expression in the trophoblast may play a key role in parturition and initiation of labor in pregnant human females.

## Introduction

Human pregnancy lasts for approximately 38 weeks after conception. The timing of birth in mice is closely related to maturation of the fetal lungs [1]. In contrast, in humans, the timing of birth is associated with the development of the placenta [2]. Complex interactions between various endocrine and paracrine factors are involved in the onset of human parturition. Although there are studies that have reported on the differences in gene expression in fetal membranes between labor and non-labor group [3], the placenta seems to be an important source of these complex interactions [4]. However, the changes that occur in the placenta during labor are not well characterized.

Several studies have shown that the timing of birth is associated with levels of maternal plasma corticotropin-releasing hormone (CRH), which is of placental origin [5,6]. Increasing evidence suggests that human parturition is associated with activation of inflammatory processes in the uteroplacental unit [7,8]. Nevertheless, the precise mechanisms underlying labor have not been completely characterized.

Villous placenta consists of a heterogeneous mixture of cell types including the trophoblast layers (syncytiotrophoblasts and cytotrophoblasts) and villous core (villous stroma and fetal blood vessels). Although histological techniques such as immunohistochemistry and in-situ hybridization provide information about the localization of proteins and mRNAs encoding specific genes, these are semi-quantitative methods. To obtain meaningful results about cell function within tissues, it is important to be able to investigate pure populations of cells. Techniques to obtain pure populations of cells include cell culture, flow cytometry, and various microdissection methods [9].

Laser capture microdissection (LCM) enables the user to isolate a small number of cells or tissues from frozen or formalin-fixed, paraffin-embedded tissue sections. LCM technique allows the selection of cells with a minimum resolution of several microns. DNA, RNA, protein, and lipid components can be isolated from microdissected samples and analyzed. Chan et al. demonstrated that LCM can be used to precisely locate and accurately quantify mRNA expression in specific cell populations from placental tissue [10].

Trophoblasts are fetal-derived cells of epithelial origin that form the placental chorionic villi. Trophoblasts produce numerous immunomodulators that play a role in placental development and pregnancy maintenance [11–13]. However, the relationship between gene expression in specific placental tissues and the onset of term labor remains unclear.

We hypothesized that alterations in the expression of specific genes in trophoblasts may cause the initiation of labor. LCM allows gene expression to be studied in the trophoblast layer separately from the surrounding heterogeneous placenta. We used LCM to compare the expression of genes in the trophoblast layer of term placentas between the labor group and non-labor group. While LCM facilitates gene expression studies of the trophoblast layer, the limited amount of tissue that can be obtained limits the number of genes that can be examined.

We previously reported that there were 15 categories of differentially expressed genes when placentas of a labor group were compared with placentas of a non-labor group based on microarray analysis [14]. As *CRH, FLT-1, NF-kB1 and TLR8* were shown to be differentially expressed in the previous microarray study, we want to characterize the expression of these genes in the trophoblast layer. A recent study reported *SIRT1* gene expression was significantly lower in placenta after labor compared to before labor [15]. *SIRT1* possesses anti-inflammatory actions. *KAP1*, which is officially known as TRIM28, is a negative regulator of *SIRT1* in E2F transcription factor network. Therefore, we also examined the expression of *SIRT1* and *KAP1*. In gestational tissue from women who delivered after labor, *IL-1* and *IL-6* have been shown to be expressed at higher levels compared with tissues from women who delivered without labor [16]. As the limited amount of tissue after LCM limits the number of genes that can be studied, we examined the expression of *CRH, FLT-1, NF-kB1, TLR8 , KAP1, SIRT1, IL-1B and IL-6* in this study.

## Materials and Methods

### 2.1: Patients and sample collection

We recruited healthy women with uncomplicated pregnancies from the department of Obstetrics and Gynecology, CHA Gangnam Medical Center, CHA Medical University (Seoul, Korea). Six women with spontaneous onset of labor who delivered vaginally at term after an uncomplicated pregnancy were recruited. In addition, six women who delivered by elective Cesarean section (CS) at term were recruited. Elective CS was defined as a planned CS performed in the absence of signs of labor or rupture of membranes. All women were healthy Koreans and had low-risk pregnancies without complication such as preeclampsia, gestational diabetes, and anemia. Patients with chorioamnionitis or fetal infections were excluded from the study.

Fresh placentas were obtained immediately after delivery. Tissue samples were excised from the middle cross section (approximately 3 cm beside the umbilical cord insertion, from the middle layer of the placenta midway between the maternal and fetal surfaces). Tissue samples were collected from macroscopically normal areas, avoiding sites of infarction, hemorrhage, and fibrin deposition. The collected specimens were placed in an aseptic plastic cup, covered, and transported to the pathology laboratory within 30 minutes of delivery. The specimens were stored in a polystyrene box filled with ice during transportation.

Clinical characteristics of the mother (maternal age, parity, body mass index, antenatal steroid administration, fever during delivery) and her baby (birth weight, Apgar scores 1 & 5 minutes after birth) were collected. This study was approved by the Ethical Review Board for Human Genome Studies at CHA Gangnam Medical Center, College of Medicine CHA University (No. PKC10-040). Written informed consent (as outlined in PLOS consent form) was obtained from all participating women and husbands. A clinical summary of the patients who participated in this study is provided in Table 1.

**Table 1:** Clinical characteristics of the pregnant mothers from which the placentas were obtained.

	Group		
Characteristics	Non-labor (n=6)		Labor (n=6)
Maternal age (years)	30.5 ± 3.1		30.2 ± 2.6
Maternal BMI (kg/m^2^) ^a^	18.9 ± 2.4		18.6 ± 0.8
Gestational age at birth (days)	274.5 ± 5.4		272.5 ± 9.2
Primiparity	5 (83%)		5 (83%)
Spontaneous labor onset	No		Yes
Delivery mode	C-section		Vaginal
Birth weight (g)	3305 ± 248		3241 ± 243
Baby’s sex (female / male)	6 / 2		6 / 2
Apgar - 1 min	8 (7-8)		8 (7-8)
- 5 min	9 (9)		9 (8-9)
Labor duration (hours)	0		6.7 ± 0.8

BMI = body mass index.

Data presented as the means (± SD), medians (range) or n (%).

a Based on first antenatal visit at approximately 12 weeks of gestation.

### 2.2: Specimen preparation

Under RNase-free conditions, a small segment of the delivered placenta was cut and embedded in Tissue Tec optimal cutting temperature (OCT) compound. It was then rapidly frozen in a HM525 cryostat and 6 μm-thick frozen sections were cut serially. The sections were placed on special membrane-coated slides (PEN Membrane Glass Slide LCM0522; Arcturus). Thereafter, the slides were stored at -80°C until further processing.

### 2.3: Laser capture microdissection (LCM)

Cryosections of fresh frozen tissue on the membrane-coated slides were defrosted at room temperature. Sections were immediately fixed in 75% ethanol for 30 seconds, rinsed with DEPC-treated distilled water, and stained with hematoxylin (1 min). They were then left to dehydrate in 75% ethanol (30 sec), 95% ethanol (30 sec), 100% ethanol (30 sec), fresh 100% ethanol (150 sec), followed by xylene (5 min, twice). Slides were air-dried on the bench before LCM.

LCM was performed with a Pixcell II Laser Capture Microscope (Arcturus, Mountain View, CA). Sections were viewed using a 20X lens. A laser beam (size 30 μm, power 30 mW, duration 10 msec, 0.240 V, current 4.6 mA, temperature 24°C) was directed at selected cells that were then captured on an RNase-free cap with a transfer film (CapSure LCM cap, Arcturus). Using a laser beam, trophoblast cells (which were intermingled syncytiotrophoblasts and cytotrophoblasts) were affixed to the capture film by a brief laser pulse. Target cells were seperated and removed from the surrounding area.

### 2.4: RNA extraction

Total RNA was extracted from trophoblast cells captured by the LCM transfer film using a PicoPure RNA Isolation Kit (Arcturus). Captured cells were incubated with 50 μl of extraction buffer at 42°C for 30 min, mixed with 50 μl of 70% of ethanol, and pipetted into a purification column. After the purification column was centrifuged and washed several times, total RNA was eluted for reverse transcription.

### 2.5: Reverse transcription

Total RNA was extracted with Trizol reagent (Gibco BRL, Grand Island, NY, USA) from pooled trophoblast layer cells collected from the placentas of subjects according to the manufacturer’s instructions. The RNA concentration (nanograms per microliter) of each sample was measured using a NanoDrop^®^ ND-1000 spectrophotometer (Nanodrop Technologies Inc., USA). For LCM samples, cDNA was synthesized from extracted RNA using a SuperScript^®^ III First-Strand Synthesis system (Invitrogen, Grand Island, NY, USA). Briefly, after incubation of total RNA with 1 μl of oligo(dT)^20^ primer and 1 μl of dNTP at 65°C for 5 min, the reaction was carried out in 25 mM MgCl_2_ and 0.1 M DTT mix in a final volume of 40 μl. Two hundred units of reverse transcriptase and 40 U RNase OUT were added. Reverse transcription was conducted at 50°C for 50 min, and the reaction was then terminated by incubation at 85°C for 5 min to inactivate the enzymes. After addition of 2 units of RNase H, the mixture was incubated for 20 min at 37°C to cleave the RNA template and stored at -20°C until use. 

### 2.6: RT-PCR and Real-time RT-PCR

Primer pairs used in this study are described in Table 2. To validate the expression of the selected genes, we performed RT-PCR and real-time RT-PCR. The standard RT-PCR was used as a preliminary experiment for gene expression and quantitative analysis. Differences in gene expression between the two groups are quantitatively analyzed using real-time RT-PCR. *28S* rRNA expression was used to normalize the data. PCR reactions contained 500 ng of cDNA, PCR buffer, 200 µM each of dNTP, 10 pmol of primers, 1.5 mM MgCl_2_, and 1.5 units *AmpliTaq Gold* ^®^ polymerase (Applied Biosystems, Foster city, CA) in a final volume of 25 µl. PCR amplification was performed in a thermocycler (MJ research, INC., USA) with an initial heating step at 95°C for 10 min followed by 35 cycles of 95°C for 30 sec, 60°C for 30 sec, 72°C for 30 sec, and a final elongation step at 72°C for 10 min. The amplified products were analyzed by gel electrophoresis. Real-time RT-PCR was performed in a 96-well plate using an IQ5 icycler (Bio-Rad, Hercules, California, USA). The 20 μL reaction mixtures contained 20 ng cDNA, 50 nM primers, and DyNAmo™ Flash SYBR^®^ Green qPCR kit reagents (Thermo Fisher Scientific Inc., Finland). The initial cycling conditions were a 10 min polymerase activation step at 95°C followed by 40 cycles of 95°C for 30 sec, 60°C for 30 sec, 72°C for 30 sec, and a dissociation step. The results were analyzed by the 2^−ΔΔCt^ method based on cycle threshold (Ct) values using *18S* cDNA as an internal control. First, the ΔCt was calculated by subtracting the average Ct value of *18S* cDNA from the average Ct value of the target gene, and then the ΔΔCt value was calculated by subtracting the ΔCt values of the control (non-labor) group from the labor group. Fold differences were calculated as 2^-ΔΔCt^. 

**Table 2:** Sequences of oligonucleotide primers used to amplify target genes and expected RT-PCR product sizes.

Targeted gene	Location	Primer sequence (5’ → 3’)		Annealing Tm(℃)	Size(bp)
SIRT1	Exon 7	ForwardReverse	TGAATATGCCAAACTTTGCTG GGGTGGCAACTCTGACAAAT	60	103
KAP1	Exon 13	Forward Reverse	CTAGTGGCAGCACCAGCTC GCAAATGGTGGCACTGTCAT	63	101
CRH	Exon 2	Forward Reverse	CTTCGCCGGAACAGGCGACC CCGAGGGCATTCCTAGCGCC	63	121
IL-1B	Exon 5	Forward Reverse	CGATGCACCTGTACGATCAC TCCATATCCTGTCCCTGGAG	60	114
TLR8	Exon 2	Forward Reverse	TGGTTTGCCAGAGTCTTTGA GCAGTTCCAGGCCAAATAGA	60	118
FLT1	Exon 15	Forward Reverse	TTAGGACCAGGAAGCAGCAC TGCTGAACTTTCCACAGAGC	60	108
IL-6	Exon 3	Forward Reverse	AGACTTGCCTGGTGAAAATCA ACAGCTCTGGCTTGTTCCTC	60	103
NF-kB1	Exon 24	Forward Reverse	GAGACATCCTTCCGCAAACT GTCCTTCCTGCCCATAATCA	60	100
28s rRNA		Forward Reverse	TTGAAAATCCGGGGGAGAG ACATTGTTCCAACATGCCAG	60	100

### 2.6: Statistical analysis

Each experiment was repeated at least three times and mRNA expression was quantified by densitometric scanning. Student’s *t*-test was used to compare mRNA expression between two groups for each gene. A value of *p*<0.05 was considered to be statistically significant. The number of asterisks indicates the p-value (*, *p*<0.05).

## Results

Six pregnant women were recruited to each of the labor and non-labor patient groups. There was no significant difference in maternal age, parity, gestational weeks, maternal body mass index, or Apgar scores of the babies between the two groups (Table 1). The only significant difference was that the labor duration in the labor group ranged from 6 hours to 8 hours but was 0 hours in the non-labor group. Four women in the labor group were administered an epidural analgesia for pain relief. None of the women had underlying disease or fever during delivery.

**Figure 1:**
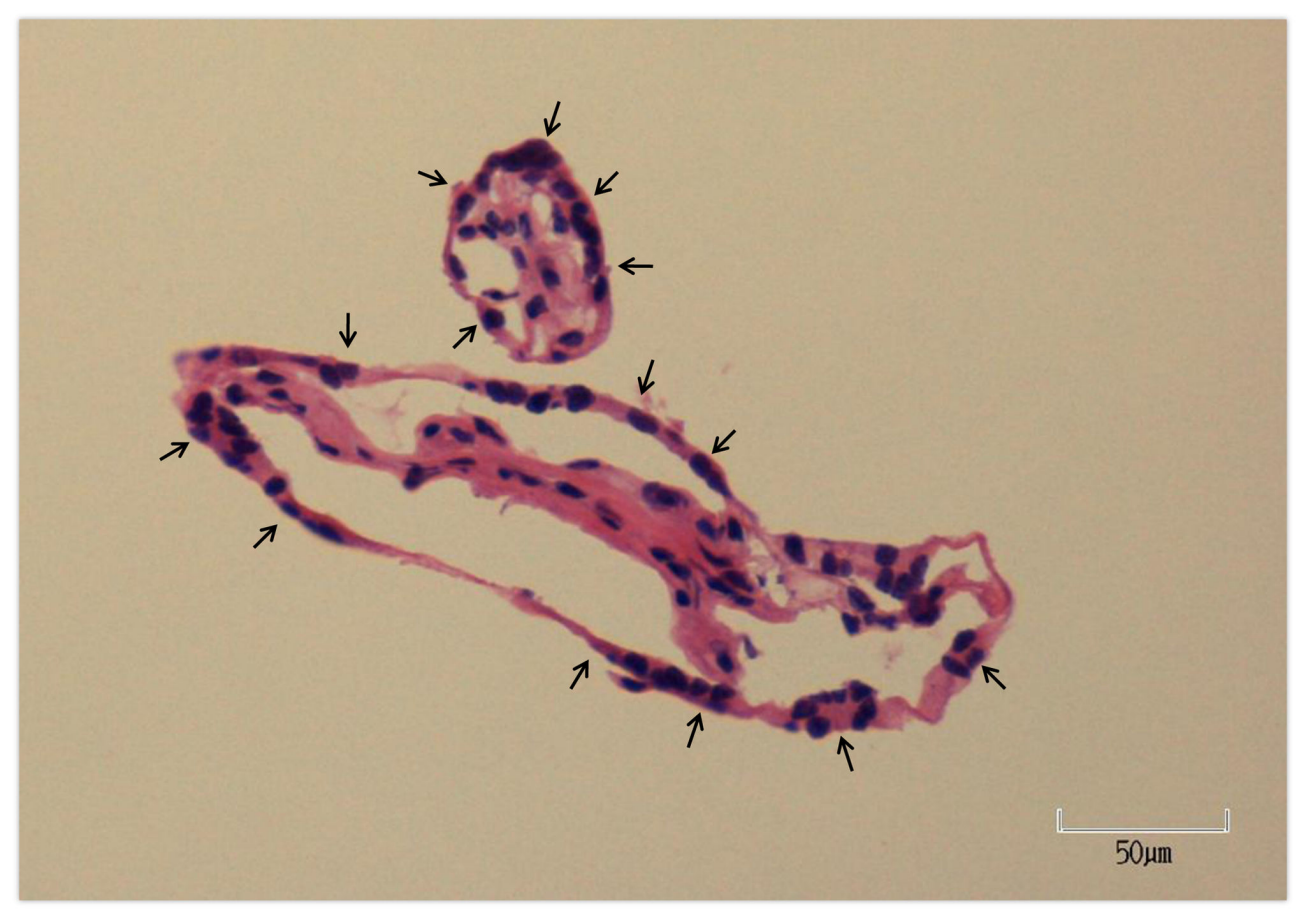
H&E staining of villous placenta. This is a H&E stained slide of normal term placenta shown at 20X magnification. Arrows indicate trophoblast layer.

**Figure 2:**
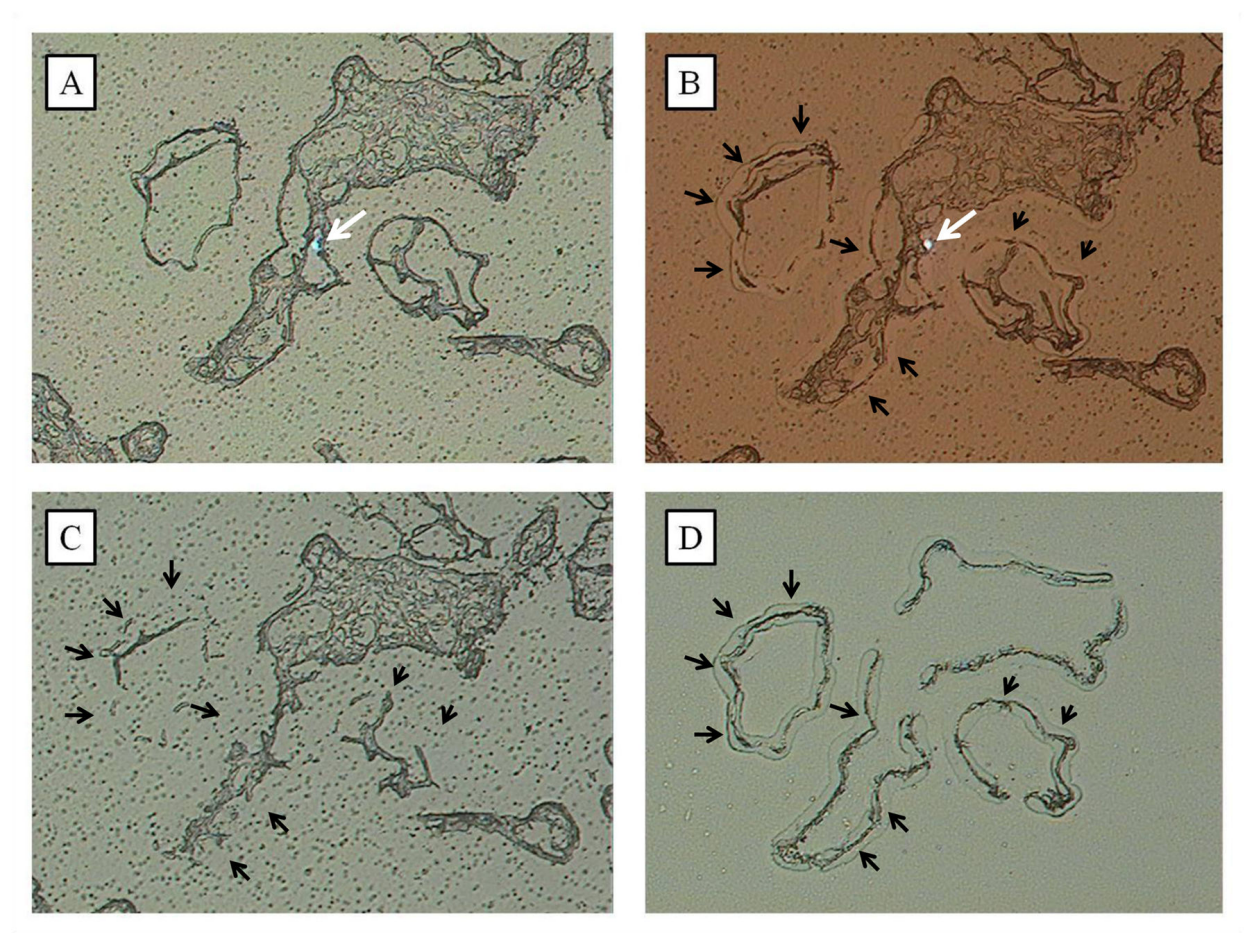
Process of laser capture microdissection. A, B, and C are sections of normal term placenta shown under laser capture microscope. A is a photograph showing chorionic villi with a laser spot (white arrow) in the middle. B is the same area showing trophoblasts (black arrows) affixed to the capture film by a laser pulse. C is the same area showing tissue left behind on the slide after laser capture of the trophoblast cells. D is an image of the cap with transfer film that was affixed to the trophoblasts (black arrows).

A representative placental specimen is shown in Figure 1. The laser capture microdissection technique used to capture trophoblasts is shown in Figure 2. The trophoblast layer of the chorionic villi was cut by laser and collected for further study. One LCM cap contained trophoblasts pooled from approximately 500 chorionic villi. Five separate caps of trophoblasts were obtained from each placental sample. The mean RNA amount per LCM cap was 130.5 ± 10.7 ng in the labor group and 129.3 ± 10.9 ng in the non-labor group.

**Figure 3:**
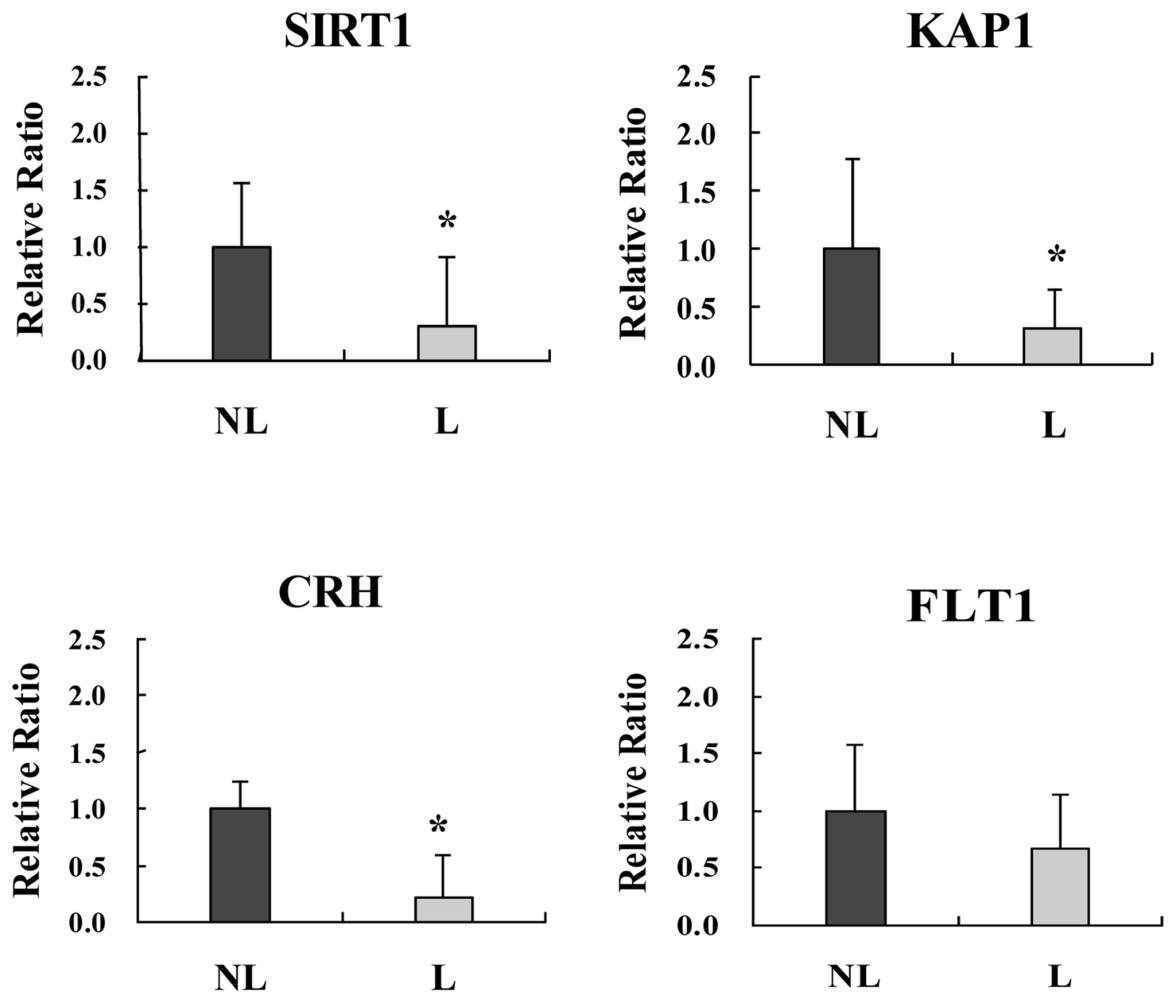
Real-time RT-PCR results. Graphic representations of real-time RT-PCR data for SIRT1, KAP1, CRH and FLT1. The data were normalized to 28S rRNA expression. The data are reported as means ± SD with significant differences between the non-labor group and the labor group labeled with asterisks (*, *p*<0.05). NL and L represent non labor placenta and labor placenta, respectively (n=6).

There was significantly less mRNA encoding *SIRT1* (*p*=0.0244), *KAP1* (*p*=0.0390) and *CRH* (*p*=0.0288) expressed by trophoblasts from the labor group than the non-labor group (Figure 3). The expression of *FLT*-1 (*p*=0.2891) was not significantly different between the two groups.

**Figure 4:**
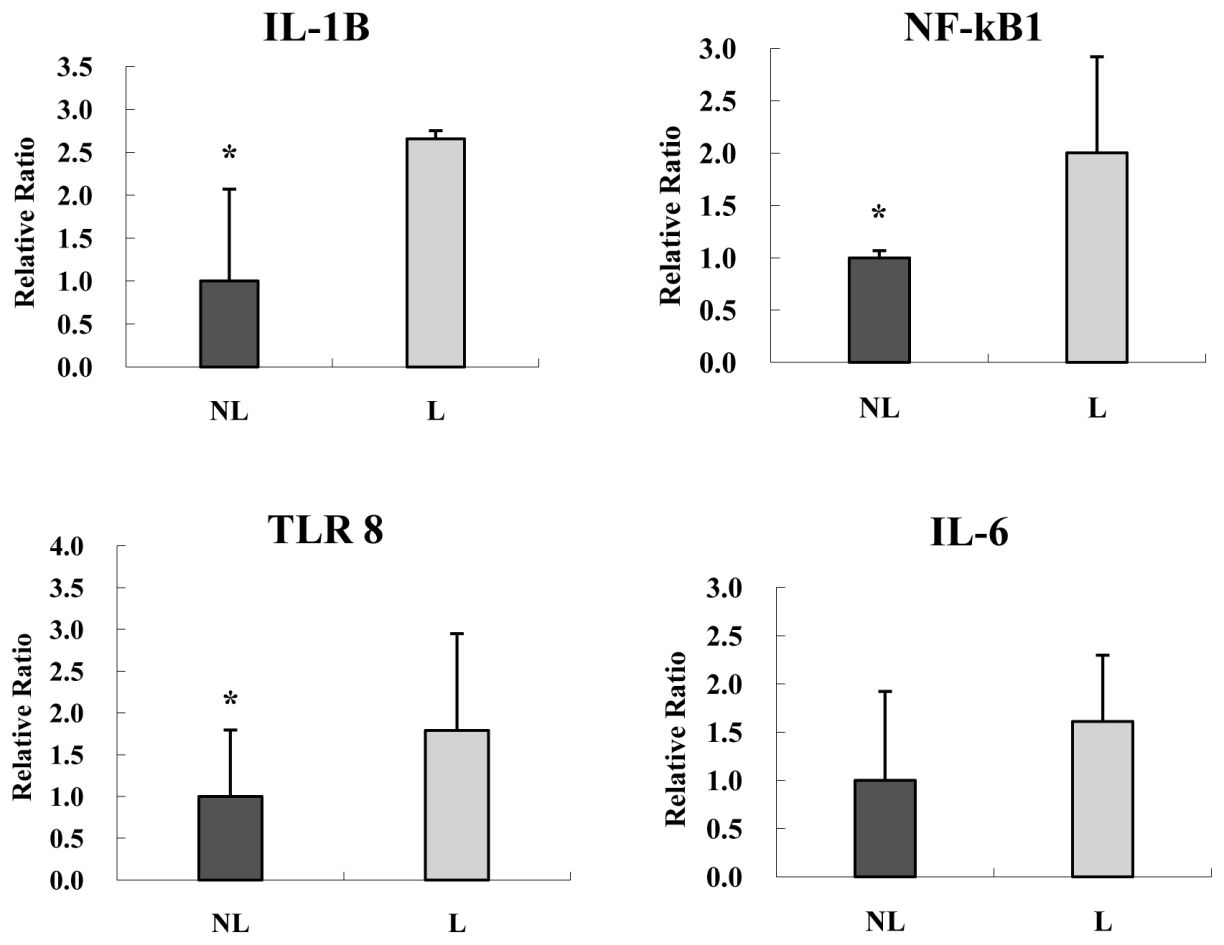
Real-time RT-PCR results for genes associated with inflammation. Real-time RT-PCR results for genes associated with inflammation in the trophoblast layer of labor placenta. The data are reported as means ± SD with significant differences between the non-labor group and the labor group labeled with asterisks (*, *p*<0.05) NL and L represent the non-labor and labor group, respectively (n=6).

The expression of *IL*-1*B* (*p*=0.0372), *NF-kB1* (*p*=0.0283) and *TLR 8* (*p*=0.0310) in the labor group was significantly higher than that in the non-labor group (Figure 4). Although increased expression of *IL-6* was observed in the labor group, it is not statistically significant (*p=0.3038*).

## Discussion

This is first study to use laser capture microdissection (LCM) to isolate trophoblasts from normal human placenta tissue to compare trophoblast mRNA expression between women who underwent spontaneous labor and delivered vaginally and those who did not go into labor and delivered by C-section. Researchers from our center previously reported that 351 genes were differentially expressed in the placentas of a vaginal delivery group compared to the placentas of a Cesarean section group. These genes appeared to be closely associated with the inflammatory response that characterizes labor [14].

*IL-1* and *-6* and *TNF-* α have been shown to be expressed at higher levels in gestational tissue after preterm and term labor compared with tissues from women who delivered at similar gestational ages without labor [16]. Genomic analysis of labor versus non-labor uterine and fetal tissue has confirmed that inflammatory genes are among those genes whose expression is most profoundly altered during labor [17,18]. Normal term labor is associated with upregulation of inflammatory pathways due to *nuclear factor-kappa B* (*NF-kB*) activation and functional progesterone withdrawal [19]. Inflammation may induce parturition by activation of inflammatory stimuli signaling via Toll-like receptors (TLRs), which results in prostaglandin (PG) and matrix metalloproteinase (MMP) production in addition to leukocyte invasion into reproductive tissues, culminating in myometrial contractility, rupture of membranes, and cervical ripening [20]. Thus, *NF-kB*, possibly subsequent to activation of *TLRs*, might play a critical role in signaling pathways that increase inflammatory cytokine production. In the current investigation, *IL-1B*, *NF-kB1*, and *TLR 8* were significantly upregulated in the trophoblast layer of placentas from women who went into labor spontaneously. This is consistent with earlier findings [14,16]. There was no statistically significant difference for IL-1B in previous microarray study, but the fold change for IL-1 receptor 1 (IL-1R1) was 4.27 [14]. The reason having the different results in the two studies are because the previous microarray study used mixed placental tissue but this LCM study was conducted only using only the trophoblast layer. This is why this study is important. Although increased expression of *IL-6* was observed in the labor group, the data collected in our study is statistically insignificant. It is very likely caused due to a small sample size. Further study with a larger sample size is required to clarify this.

In the plasma of non-pregnant women, the concentration of CRH is around 15 pg/ml. During pregnancy, however, the mean plasma CRH level increases to approximately 800 pg/ml during the late third trimester and becomes undetectable within 24 hours after delivery [21]. Circulating levels of placental-derived CRH increase exponentially as human pregnancy progresses towards term, which has led to the suggestion that CRH may act as a ‘placental clock’, determining the length of pregnancy [22]. *CRH* mRNA is largely localized in syncytiotrophoblasts and intermediate trophoblast bodies [23]. One study demonstrated that expression of *CRH receptor type I* in upper segment human myometrium was reduced during labor [24]. In our study, we found that expression of *CRH* in the trophoblast layer was lower in the labor group than in the non-labor group. The level of CRH increases as human pregnancy progresses towards term and then decreases rapidly after delivery. Collectively, these data suggest that spontaneous labor is associated with downregulation of *CRH* expression in the trophoblast layer. It is estimated that the postpartum reduction of plasma CRH precede by a decrease in RNA expression of trophoblast before or during spontaneous labor.

**Figure 5:**
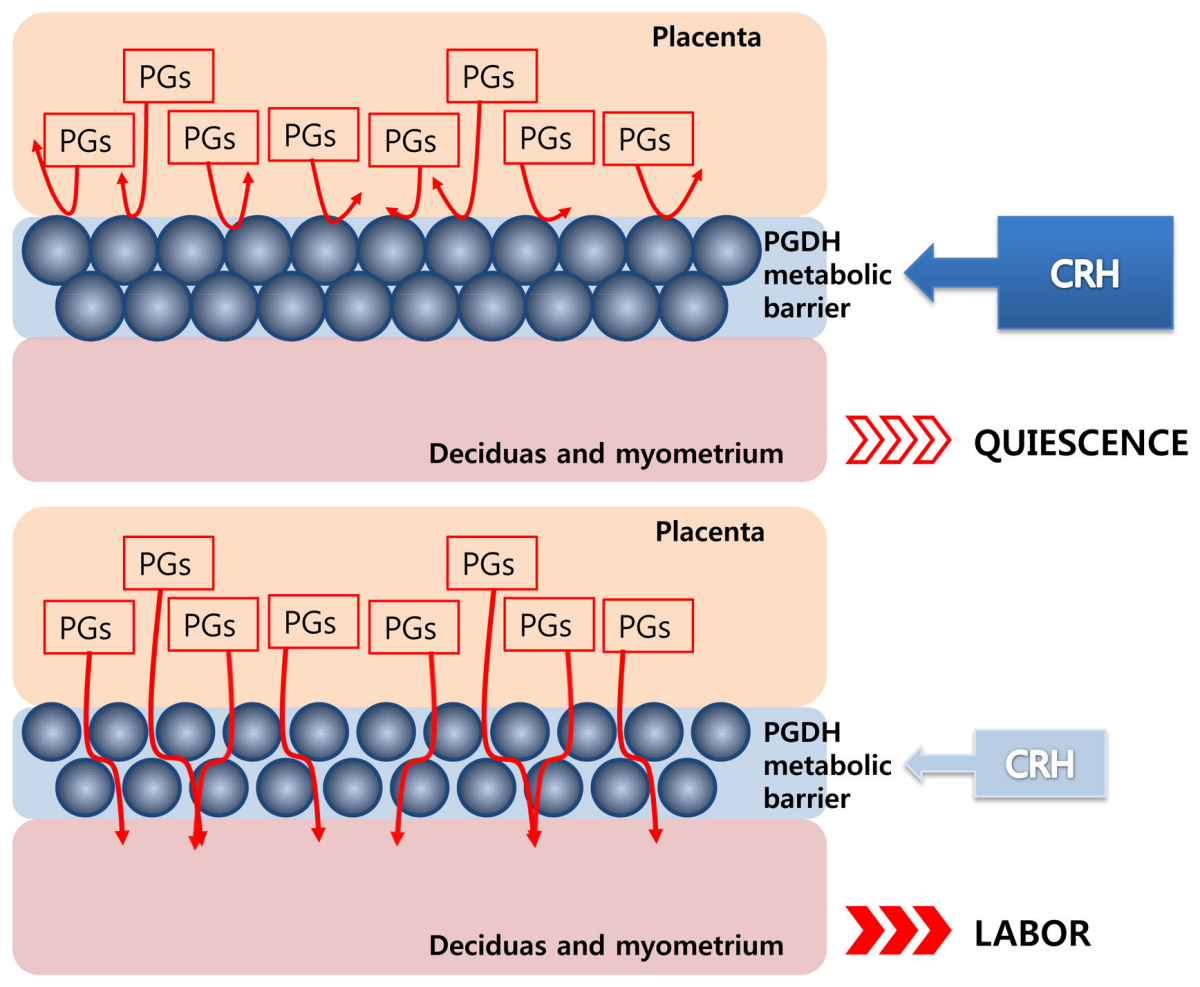
A schematic illustration representing the action of CRH in the placenta. A schematic illustration of representing the central action of CRH in the placenta. A) High level of CRH stimulates or maintains PDGH activity and acts as a metabolic barrier preventing prostaglandins (PGs) from crossing into the deciduas and myometrium. B) Low levels of CRH do not stimulate or maintain PDGH activity. Therefore, PGs can cross into the deciduas and myometrium, where they cause uterine contraction and labor.

At the onset of term and preterm labor, there is a large increase in the amount of active prostaglandins (PGs) [25]. PGs play a crucial role in mediating parturition events by stimulating uterine contractility and causing ripening of the cervix [26,27], and their synthesis and metabolism are regulated predominately by 15-hydroxy-PG dehydrogenase (PGDH) within chorionic tissue [28,29]. PDGH located within the chorion has been suggested to play a crucial role in maintaining a metabolic barrier to bioactive PGs, preventing PGs from crossing into the deciduas and myometrium, where they could cause early uterine contraction [29,30]. One study suggested that CRH may act to stimulate or maintain PDGH activity in trophoblast cells and may help maintain a metabolic barrier, preventing the transfer of bioactive PGs from the placenta to the myometrium [31]. In our study, the expression of *CRH* in trophoblast cells was significantly lower in the labor group than the non-labor group. We suggest that downregulation of *CRH* in trophoblast cells may be one of the causes of labor onset via perturbation of the metabolic barrier (Figure 5).

Sirtuins(SIRT) are enzymes that catalyze NAD^+^-dependent deacetylation and/or ADP-ribosylation of target proteins, and have been implicated in aging, cell death/survival, metabolism, stress resistance, and endocrine signaling [32]. There are seven human isoforms (SIRT1-SIRT7) of SIRT that differ in their subcelluar localization, tissue distribution, and protein substrates. *SIRT1* expression has been reported to be significantly lower in the placenta of women who underwent spontaneous labor than those who did not go into spontaneous labor. The SIRT1 activators resveratrol and SRT1720 significantly decrease LPS-induced *TNF*, *IL-6* and *IL-8* gene expression and repress the mRNA expression of the time-limiting enzyme involved in prostaglandin formation [15]. *SIRT1* is a negative regulator of proinflammatory cytokines [33,34] and *MMP9* expression [35] and has anti-inflammatory actions. A previous study showed that in term placenta, SIRT1 staining was localized to the syncytiotrophoblast layer, while there was minimal staining in cytotrophoblasts [15]. KAP1, also known as TRIM28, is negative regulator of SIRT1 in E2F transcription factor network pathway. In our study, levels of *SIRT1* and *KAP1* were significantly downregulated and levels of *IL-1B*, *NF-kB1*, and *TLR 8* were significantly upregulated in the labor group compared to the non-labor group. Collectively, these data suggest that downregulation of *SIRT1* causes downregulation of *KAP1* expression through negative feedback. Decreased expression of *SIRT1* causes decrease of anti-inflammatory actions, which induces the expression of inflammatory cytokines and increases myometrial contractility.

A microarray analysis study using human placenta reported elevated *FLT* expression in the placentas of mothers that had undergone spontaneous labor versus controls [14]. In contrast to this previous study, we found that the expression of FLT-1 was not significantly increased in the labor group in our study. Previous study used placental tissue which consisted of a heterogeneous mixture of cell types. In our current study, only trophoblasts were dissected from placental tissue. Further research is required to clarify this discrepancy. 

To the best of our knowledge, this is the first study to compare gene expression in the LCM-obtained trophoblast layer of placental tissue from mothers who gave birth spontaneously via vaginal delivery (labor group) and those who delivered their child via Cesarean section (non-labor group). One limitation of our study is that the sample number was small, but LCM is laborious and time-consuming work. Second, the modes of delivery differed between the two groups. To overcome this limitation, it will be necessary to compare placental gene expression changes after Cesarean section involving labor and Cesarean section not involving labor. The third limitation is that the changes in gene expression we observed may be a cause of labor onset and progress, or simply an effect of labor.

In summary, when we compared gene expression in the trophoblast layer of the placenta between a vaginal delivery group that underwent labor and a Cesarean section group that did not undergo labor, we found that *SIRT1*, *KAP1*, and *CRH* expression was downregulated and *IL-1B*, *NF-kB1*, and *TLR 8* were upregulated in the trophoblast layer of placentas in the labor group. Downregulation of *SIRT1*, *KAP1*, and *CRH* expression in the trophoblast may play a key role in parturition and initiation of human labor. Specifically, human term labor may be induced by decreased expression of *SIRT1*, *KAP1*, and *CRH* in the trophoblast layer of the placenta and this may be closely associated with the inflammatory response. In this study, we used a novel approach to study gene expression in placental tissue. An understanding of gene expression alterations in specific placental tissues during labor will provide new opportunities to determine the precise mechanisms that regulate parturition.
